# Comparative seed dormancy and germination responses to drying and flooding in two Sri Lankan wild rice species

**DOI:** 10.1093/aobpla/plag034

**Published:** 2026-08-11

**Authors:** T Sajeevan, Malaka M Wijayasinghe, K M G G Jayasuriya, A Corli, Fiona R Hay, A Mondoni

**Affiliations:** Department of Biological Sciences, Faculty of Applied Sciences, South Eastern University of Sri Lanka, Sammanthurai 32 200, Sri Lanka; Department of Earth and Environmental Sciences, University of Pavia, Pavia 27 100, Italy; Department of Biological Sciences, Faculty of Applied Sciences, Rajarata University of Sri Lanka, Mihintale 50 300, Sri Lanka; Department of Botany, Faculty of Sciences, University of Peradeniya, Peradeniya 20 400, Sri Lanka; Department of Earth and Environmental Sciences, University of Pavia, Pavia 27 100, Italy; Department of Agroecology, Aarhus University, Forsøgsvej 1, Slagelse 4200, Denmark; Department of Earth and Environmental Sciences, University of Pavia, Pavia 27 100, Italy

**Keywords:** crop wild relatives, dormancy cycling, dry after-ripening, *in situ* conservation, *Oryza*, rice, seed dormancy

## Abstract

Sri Lankan wild rice, *Oryza nivara* and *Oryza rhizomatis*, are two threatened species occurring in habitats with contrasting dry and wet seasonal patterns. Both species produce dormant seeds at dispersal, yet their germination phenology remains poorly understood. This study aimed to elucidate their germination strategies and dormancy mechanisms to support conservation efforts. We hypothesized that dormancy alleviation in these species is niche-specific; specifically, that *O. rhizomatis* requires longer periods of dry storage (after-ripening) and shorter period of flooding than *O. nivara*. (i) Freshly harvested seeds of both species were subjected to nine dry-storage periods (0, 2, 4, 6, 8, 10, 12, 18, and 20 months) at 25°C and 60% relative humidity (RH). Following storage, seeds were incubated on moist tissue paper for 30 days at 25°C under a 12 h light/dark regime. Additionally, a second set of seeds stored for 6, 8, 18, and 20 months was exposed to flooding treatments (FTs) (1, 2, or 4 weeks) prior to incubation. (ii) In both species, germination percentage increased up to ∼80% with increasing dry-storage periods up to 12 months. Under prolonged dry storage, germination percentage declined in *O. rhizomatis* and none of the seeds germinated in *O. nivara*. In *O. rhizomatis*, a short FT (1 week) increased the germination percentage compared with dry storage alone, whereas longer flooding periods (2–4 weeks) reduced it. In contrast, flooding had no significant effect on seed germination in *O. nivara*. (iii) Overall, prolonged drying effectively breaks dormancy in both species, preventing germination immediately after dispersal when drought risk is high and seedling survival is low. The species exhibit distinct physiological dormancy breaking responses that likely contribute to their differing ecological and geographical distributions. Predicted increases in precipitation under climate change may constrain recruitment by limiting dry after-ripening or increasing flooding duration, posing a particularly high risk for the endemic *O. rhizomatis*.

## Teaser

This study aimed to determine the environmental cues which regulates the seed dormancy of two Sri Lankan wild rice species and to determine whether their dormancy-breaking responses reflect adaptations to their contrasting ecological habitats. *Oryza rhizomatis* required a longer period of dry after-ripening but a shorter flooding duration to achieve maximum germination compared with *O. nivara*, suggesting distinct dormancy release strategies associated with their respective environments Understanding the germination ecology and dormancy mechanisms of these species provides valuable insights for developing effective conservation, restoration, and sustainable management strategies for wild rice genetic resources.

Key message Climate-driven changes in rainfall patterns affect seed dormancy and germination in Sri Lankan wild rice species.

## Introduction

The *Oryza* genus has two domesticated species, namely *Oryza sativa* from Asia and *Oryza glaberrima* from Africa, and 22 wild rice (*Oryza*) species (Henry 2019). Wild rice species (2 *n* = 24, 48) represent the AA, BB, CC, BBCC, CCDD, EE, FF, GG, KKLL, and HHJJ genome types (Brar and Khush 2018). Sri Lanka is home to five wild rice species: *Oryza eichengeri* Peter, *Oryza rufipogon* Griff, *Oryza nivara* Sharma et Shastry, *Oryza granulata* Nees et Arn. ex Watt and the endemic, *Oryza rhizomatis* Vaughan (Liyanage et al. 2002). Although most of these species are edible (Kennedy and Burlingame 2003), they are not used as common crops due to poor seed production and early seed shattering (Abdelghany et al. 2022). However, these species serve as a reservoir of valuable genetic traits that can enhance cultivated rice. Specifically, they offer potential for improving climate adaptability, productivity, and tolerance to stressors such as salinity, drought, and pathogens (Sakai and Itoh 2010). For instance, photosynthetic rate is highest in *O. nivara* followed by *O. eichengeri* and *O. rufipogon*, which was higher than that of a selection of hybrids and cultivars of *O. sativa* (Kiran et al. 2013). Such elevated photosynthetic rates in wild rice species likely represent adaptations to native habitats, where high light intensity, fluctuating water availability, and competitive conditions favour rapid carbon assimilation and growth (Kiran et al. 2013). Additionally, *O. rufipogon* (Solis et al. 2021), *Oryza alta*, *Oryza latifolia* and *Oryza coarcta* (Prusty et al. 2018) tolerate salinity better than the cultivated rice varieties. Furthermore, *O. rhizomatis* has high resistance against rice blast disease, one of the common rice diseases (Das et al. 2012). In addition to this, *Oryza longistaminata* and *O. rufipogon* may serve as sources of novel alleles to improve leaf traits under drought stress, specifically, for maintenance of leaf elongation, stomatal conductance, and membrane stability (Liu et al. 2004). The possibility of using these traits in genetic improvement programs is directly related to the presence and abundance of these plants in nature, where it is also possible for them to continue to adapt to the local environment. However, Sajeevan et al. (2023) reported that wild rice (*Oryza*) species in Sri Lanka are threatened due to anthropogenic activities, over-grazing by animals, natural disasters and ecological competitors, emphasizing the urgent need for conservation actions. Among the Sri Lankan wild rice species, *O. rhizomatis*, *O. eichengeri*, and *O. rufipogon* were identified as high-priority species for *in situ* conservation while *O. granulata* and *O. rhizomatis* were listed as high-priority species for *ex situ* conservation (Sajeevan et al. 2023). *Oryza nivara* is not considered a high-priority species for conservation in Sri Lanka due to its widespread distribution. However, several populations face potential decline since they are often treated as weeds in rice crop fields (Weerakoon 2022).

In the context of plant conservation, the study of seed ecology provides important information for both *in situ* and *ex situ* actions (Way 2003). For instance, germination and dormancy-breaking information is necessary for species restoration and seedling production (Kildisheva et al. 2020). Furthermore, understanding seed germination behaviour is crucial to predict the optimal timing and the microhabitat conditions for plant reintroduction (Baskin and Baskin 2014). Wild rice species may exhibit varying degrees of physiological dormancy (PD, e.g. Vaughan 1994, Bellairs et al. 2015, Timple et al. 2018), which prevents the embryo from emerging through the covering layers of the seed coat due to a lack of growth potential (Baskin et al. 2017). Weerakoon (2022) reported that seeds of *O. nivara* and *O. rhizomatis* are dormant at dispersal. However, the class of dormancy of the two species has not been reported in the literature. Nevertheless, seed dormancy in wild *Oryza* species has been considered to be PD (Veasey et al. 2004, Bellairs et al. 2015, Timple et al. 2018). Dormancy-breaking treatments such as scarification, gibberellic acid treatment and pre-soaking with chemicals such as H_2_O_2_, KNO_3_ and HNO_3_ have been found to alleviate seed dormancy of several wild rice species (Waheed et al. 2012, Bellairs et al. 2015, Timple et al. 2018). For example, dry-heat treatments increased the germination (up to c. 70%), but only after exposure to KNO_3_ in *O. nivara* (Timple et al. 2018). Conversely, seed germination in *O. rhizomatis* has often been null or very low (i.e. <30%), even after exposure to several pre-treatments (e.g. H_2_O_2,_ dry or chemical treatments; Timple et al. 2018). PD in cultivated rice varieties is commonly alleviated via dry after-ripening (Du et al. 2015), a natural mechanism that controls dormancy in many species from dry climates (Finch-Savage et al. 2007). Veasey et al. (2004) studied the effect of dry storage (after ripening) on four wild rice species including *O. nivara* and *O. rhizomatis*. Short-term dry storage (e.g. 2 months) was sufficient to alleviate seed dormancy in several *Oryza* species, such as *O. latifolia*, *Oryza grandiglumis*, *O. eichengeri*, and *Oryza punctata*. However, Veasey et al. (2004) stated that other species, including *O. rufipogon*, *Oryza glumaepatula*, *O. nivara*, and *O. rhizomatis*, exhibited deeper dormancy, and no dormancy breaking treatment was identified for them. Nevertheless, these studies highlighted that dry after-ripening alone may not be adequate for breaking dormancy and suggested the presence of additional dormancy-breaking mechanisms in wild *Oryza* species.

Although seed germination has a genetic basis, several studies have shown how differences in germination requirements were related to habitat conditions (Mondoni et al. 2009, Tudela-Isanta et al. 2018). *Oryza rhizomatis* inhabits the dry zone of Sri Lanka, while *O. nivara* is found in both the dry and intermediate zones (Sajeevan et al. 2023). In both regions, prolonged dry periods followed by the rainy season are the dominant climatic pattern. These species typically grow along the edges of water bodies and paddy fields, where their seeds undergo several months of drying after dispersal. This is followed by a period of flooding during the onset of the rainy season, creating conditions necessary for germination. After the floodwaters recede, the seeds begin to germinate, suggesting that a period of dry after-ripening followed by flooding and the associated reduction in oxygen availability may play a crucial role in alleviating seed dormancy in these species. Rice varieties and species exhibit contrasting responses to oxygen availability during germination, showing either tolerance or sensitivity to hypoxic conditions depending on their genotype (Ismail et al. 2013). In recent years, increasing attention has been directed towards identifying rice varieties capable of successful germination under hypoxic conditions (Yuan et al. 2024). Such varieties provide valuable material for investigating the physiological, biochemical, and molecular mechanisms underlying hypoxia tolerance (Voesenek and Bailey-Serres 2013), offering important prospects for improving crop establishment in flood-prone environments.

In this study, we shed new light on the germination ecology of the Sri Lankan wild rice species *O. rhizomatis* and *O. nivara* to assist their *ex situ* and *in situ* conservation. In particular, we identify the natural dormancy-breaking cues of these two species by simulating the effects of different periods of dry-after ripening, flooding or both in the laboratory. As the two species are found in different habitats (Liyanage et al. 2002), we also used the information gathered in this research to test the hypothesis that seed germination behaviour is synchronized with the prevailing environmental conditions of their habitats. Thus, we hypothesized that (i) prolonged dry storage (>6 months) alleviates dormancy of both species, as they are suggested to have deep PD; (ii) *O. rhizomatis* seeds require a longer dry storage period than *O. nivara*, because they are exposed to prolonged draught in its natural environment; and (iii) incorporating flooding treatment (FT) would reduce the dry storage period required for dormancy alleviation as both are exposed to flooding in their natural habitat.

## Materials and methods

### Study species

*Oryza nivara* is an annual grass within the *O. sativa* complex. *Oryza rhizomatis* is perennial, often with adventitious roots and prominent, firm, branched underground rhizomes. The climate in the habitat of these species is seasonal with dry and wet seasons, but *O. nivara* is exposed to a longer wet season than *O. rhizomatis* according to their habitat preference and geographical distribution (Supplementary Figs. S1 and S2). The geographical distribution of *O. rhizomatis* is restricted to the dry zone of Sri Lanka (Weerakoon et al. 2018). *Oryza rhizomatis* often inhabits primary and secondary forests and open, tall scrub with grassy openings in swampy or periodically flooded areas, usually open to full sun or partial shade (Liyanage et al. 2002). Plants can be seen during the period from late December to May. In the dry zone of Sri Lanka, where *O. rhizomatis* is found, there are two consecutive rainy seasons: *Yala* and *Maha*. The *Yala* season in the dry zone occurs from mid-March to early-May and is considered the minor growing season of the dry zone because it is only effective for 2 months. Precipitation during the minor growing season is less, and the soil moisture status remains dry during this season. The major growing season of the dry zone, called *Maha*, starts with the arrival of inter-monsoon rains in mid-September/October and lasts until late January/February (Punyawardena 2010, Supplementary Fig. S1). The dry zone of Sri Lanka, where *O. rhizomatis* grows, is characterized by an annual rainfall of 850–1900 mm. Within the dry zone, *O. rhizomatis* is found in areas considered arid (Vaughan et al. 2003).

*Oryza nivara* is distributed in the dry and intermediate zones of Sri Lanka (Weerakoon et al. 2018). Plants can be seen from September to December. Habitats of *O. nivara* also receive rain during the *Yala* and *Maha* seasons (Supplementary Fig. S2). The intermediate zone receives a mean annual rainfall of 1750–2500 mm with short and less prominent wet seasons (Punyawardena 2010). Although the distribution of *O. nivara* overlaps with that of *O. rhizomatis* (Sajeevan et al. 2023), the micro-habitats of the two species are different. In contrast to *O. rhizomatis*, which occurs in natural habitats where flooding is intermittent, *O. nivara* also occurs in stagnant water such as cultivated rice fields, temporary swamps, ponds, lake shores and riverbanks (Li et al. 2006).

### Study site and seed collection

**Figure 1:**
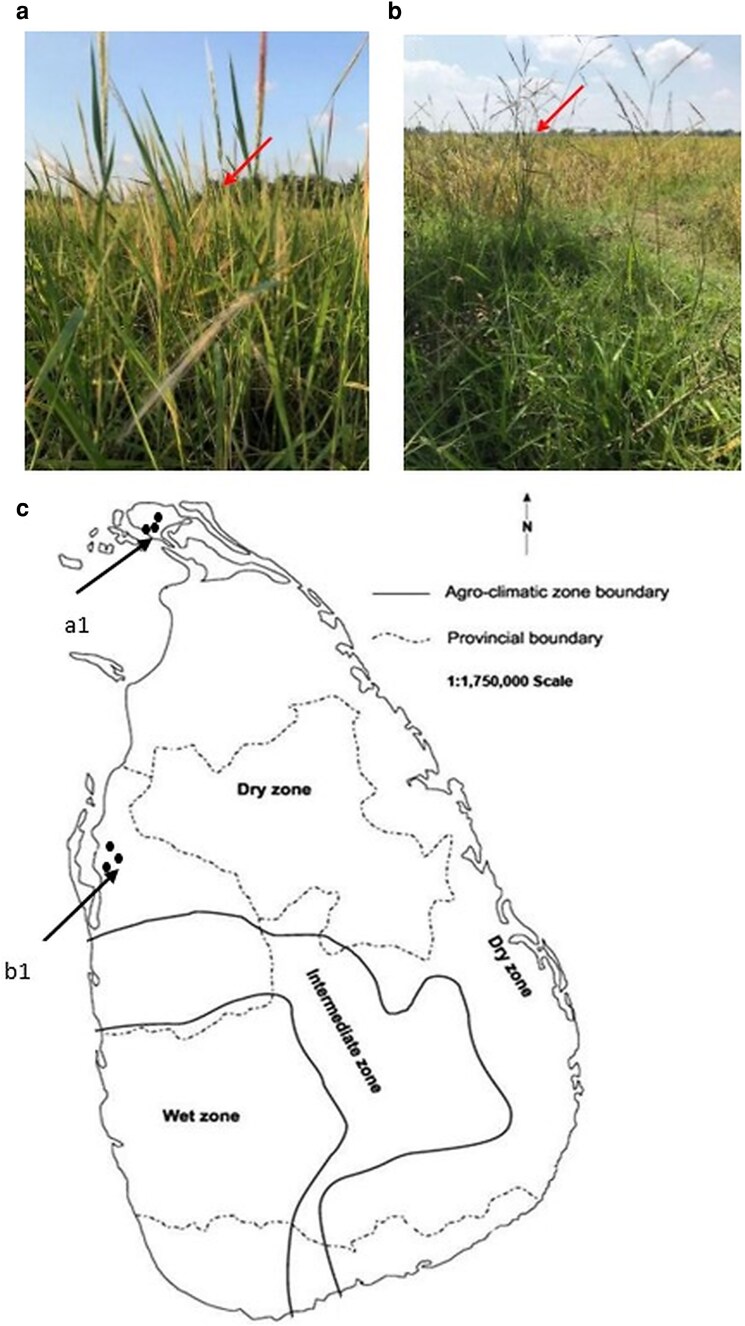
Natural habitats and collection sites of Sri Lankan wild rice (*Oryza*) species; a: *O. nivara*, b: *O. rhizomatis*. Red colour arrow indicate the wild *Oryza* species. c: Modified version of Sri Lanka map with agroclimatic zones (Geekiyanage and Pushpakumara 2013) showing Seed collection sites; a1: Seed collection sites of *O. nivara* from paddy field with stagnant water, b1: Seed collection sites of *O. rhizomatis* from frequently floodingareas in the road side and bare land.

*Oryza rhizomatis* seeds were collected from three naturally occurring sites in Karuwalagas Wewa, Puttalam district, Sri Lanka (8° 02′ 31.7″ N 79° 54′ 48.2″ E) in February 2022, while those of *O. nivara* were collected from three paddy fields in Perunkulam, Jaffna district, Sri Lanka (9° 40′ 06.2″ N 80° 09′ 37.3″ E) in March 2022 (Fig. 1). Seeds were collected from 65 *O. nivara* plants and 90 *O. rhizomatis* plants. Spikes were slightly pulled to collect only the dispersal-ready seeds and leaving immature seeds on the spike (black-coloured mature seeds vs. brown-coloured immature seeds). Seeds were collected into paper bags and brought to the seed biology laboratory, University of Peradeniya.

### Effect of dry storage on seed germination

Immediately after collection, seeds were stored in open paper bags and exposed to laboratory conditions (∼25°C, 60% RH) for 0, 2, 4, 6, 8, 10, 12, 18 or 20 months to determine whether a period of dry storage was required prior to germination. These conditions simulate those experienced by the seeds after dispersal in normal years (up to 6 months of dry season) and in dry years (up to 20 months of dry season). The 0-month consisted of fresh seeds and was considered the control treatment. After each dry period, seeds were tested for germination. Germination tests were carried our using four replicates of 25 seeds each, sowing on filter papers moistened with distilled water in 9-mm diameter Petri dishes, at 25°C in a light-controlled (12 h daily photoperiod white, fluorescent light) incubator (GHP 9270). Germinated seeds (radicle protrusion and elongation >2 mm) were counted daily for 30 days. At the completion of each germination test, non-germinated seeds were cut open and embryos were observed to check the viability (firm, white viable embryos vs. soft discoloured non-viable embryos; (Cross et al. 2020).

### Effects of flooding on the germination of fresh and dry-stored seeds

Four replicates of 25 dry-stored seeds each of both species were retrieved after 6, 8, 18, and 20 months and exposed to flooding conditions of 1, 2, or 4 weeks. FTs involved enclosing seeds in mesh bags and submerging them in 1000 ml beakers filled with distilled water, ensuring a consistent 10 cm water layer above the seeds. After each flooding interval, seeds were retrieved from the water and the number of germinated seeds was counted and recorded. Subsequently, the retrieved seeds (non-germinated) were used for germination testing, following the same methods as described above. Germination tests on dry-stored seeds without FT (0 week) were used as a control.

### Data analysis

Cumulative germination percentage after 30 days and the median germination time (*T*_50_) were used as the response variable in the data analysis. *T*_50_ was calculated using the following equation.


$$T_{50}=t_{i}+\frac{\left[\left(\frac{N}{2}\right)-n_{i}\right](t_{i}-t_{j})}{\left(n_{i}-n_{j}\right)}$$

where *N* is the final number of germinated seeds and *n_i_* and *n_j_* are cumulative numbers of seeds germinated from successive counts at times *t_i_* and *t_j_*, respectively, when *n_i_* < *N*/2 < *n_j_* (Coolbear et al. 1984). A Mann–Whitney test was performed to check the significant differences of *T*_50_ values among the treatments.

Final germination (FG, %) was modelled by fitting a generalized linear model (glm) with probit link function and binomial error (‘probit analysis’) with period of dry storage as the continuous explanatory variable; this was done using Genstat 24th Edition (VSN International Ltd). For both species, an additional ‘control viability’ parameter (*C_v_*) was included, to take into account seeds that appeared to have poor germination (Hay et al. 2014). The models were fitted separately for the two species: for *O. nivara*, excluding the data for 18 and 20 months of dry storage where there was no germination, the model covering 2–10 months of dry storage can be written as:


$$\mathrm{FG}\text{\%}=100\times C_{v}\times \Phi (g)=100\times C_{v}\times \Phi (\alpha +\beta p_{\mathrm{DS}})$$

where *Φ* is the cumulative normal distribution function, *g* is the proportion of seeds that germinate after $p_{\mathrm{DS}}$ months of dry storage, *α* represents the lag before the onset of germination and *β* represents the slope of the germination progress curve.

For *O. rhizomatis*, there was some germination after 18 and 20 months, and hence it was possible to fit the following model:


$$\mathrm{FG}\text{\%}=100\times C_{v}\times \Phi (g)\times \Phi (v)=100\times C_{v}\times \Phi (\alpha +\beta p_{\mathrm{DS}})\times \Phi \left(K_{i}-\frac{\;p_{\mathrm{DS}}}{\sigma }\right)$$

In which the additional terms are *v*, the proportion of viable seeds; *v* declines over time depending on *σ* and the theoretical initial viability of the competent seeds, *K_i_* (Ellis and Roberts 1980).

## Results

### Effect of dry storage on seed germination

**Figure 2:**
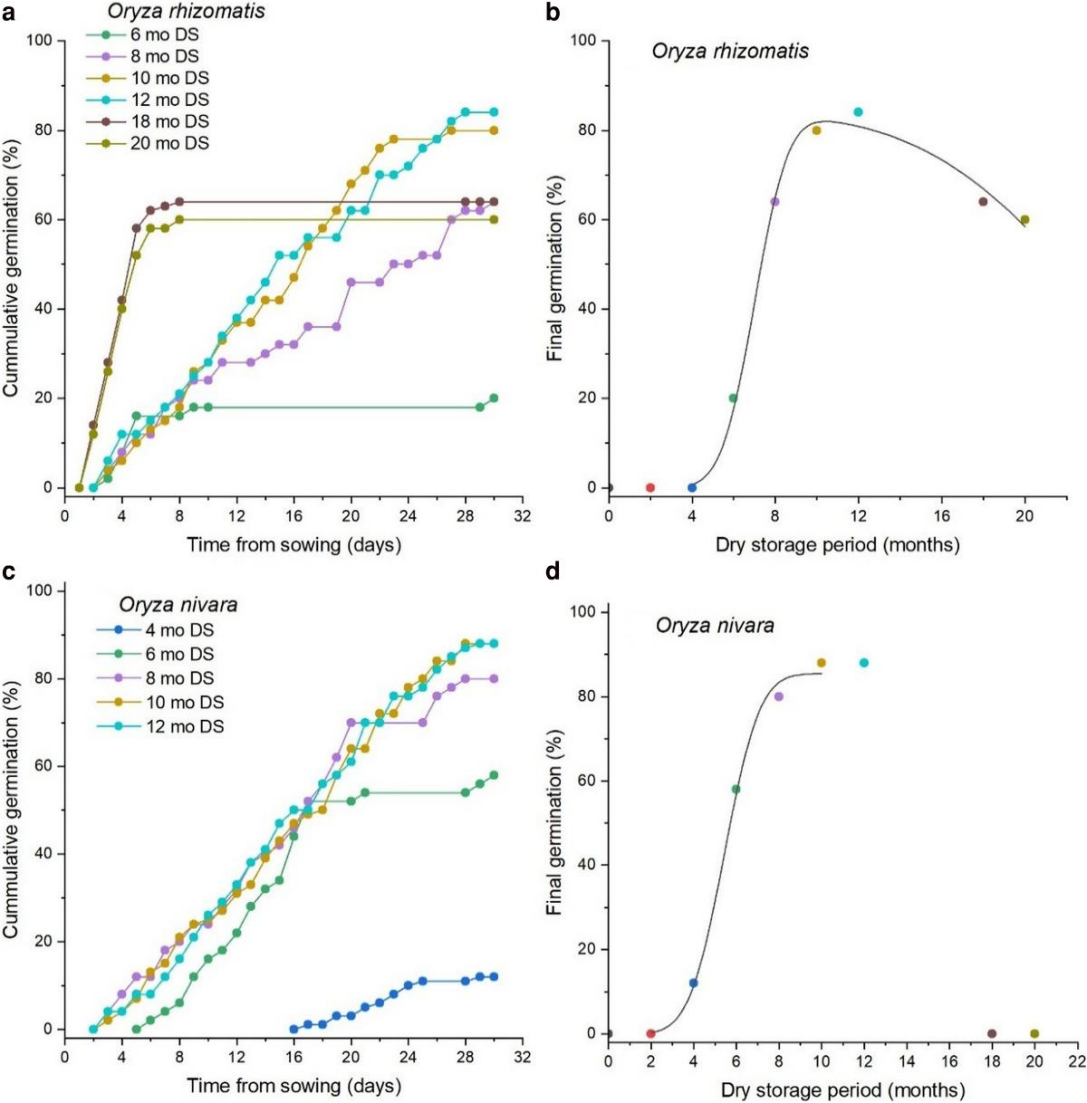
FG percentage of *O. rhizomatis* (a) and *O. nivara* (b) after 0, 8, 18, and 20 months (m) of dry storage (DS) followed by 0, 1, 2, and 4 weeks (w) of FTs and incubated on tissue paper moistened with distilled water at 25°C in light/dark (12 h/12 h) conditions. Also shown is the fraction of seeds germinating while immersed in water (under water) and the time required to reach 50% germination (red line). Different letters indicate significant differences of germination percentage at *P* < 0.05 based on GLM with Turkey's *post hoc* test (Supplementary Tables S2 and S3).

FG was significantly affected by the species, dry storage and their interaction (Supplementary Table S4). In general, germination percentage increased with increasing period of dry storage up to 12 months in both species. However, the significant interactions revealed non-consistent responses of species to dry storage. In *O. nivara*, seeds started to germinate (12%) after 4 months of dry storage and continued to increase up to 12 months (88%), whereupon none of the seeds germinated during the observation period of 30 days. *Oryza rhizomatis* required a longer drying-after-ripening period to start the germination (6 months) compared with *O. nivara*; seed germination increased with increasing drying periods up to 12 months (84%). However, further increases in dry storage period significantly reduced germination percentage to 60% (Fig. 2). The embryos of the non-germinated seeds were firm and white at the end of the germination test.

The minimal difference in *C_v_* value for *O. nivara* and *O. rhizomatis* indicates similar seed performance, serving as a consistent reference point for assessing the germination pattern under different dry storage and FTs (Table 1).

**Table 1:** Estimates from the probit model describing the increase in germination due to breaking of dormancy and decrease due to viability loss during dry storage for *O. nivara* and *O. rhizomatis*.

Species name	*C_v_*	*α*	*β*	*K_i_*	*σ*
*O. nivara*	0.85 (0.03)	−4.18 (0.54)	0.77 (0.11)	–^a^	–^a^
*O. rhizomatis*	0.84 (0.13)	−5.36 (0.77)	0.75 (0.12)	1.76 (0.34)	−0.08 (0.02)

*C_v_*, control viability parameter; *α*, lag before the onset of germination; *β*, slope of the germination progress curve; *K_i_*, theoretical initial viability of the competent seeds; *σ*, standard deviation of seed deaths in dry storage.

^a^Not estimated.

### Effects of flooding on the germination of fresh and dry-stored seeds

When FT was included in the model, the FG percentage was significantly affected by species, dry storage (as in Model 1) and by flooding time (Supplementary Table S5). Because of the strong species × dry storage and species × flooding interaction, a species-, dry storage- and flooding-specific analyses were performed. This analysis revealed that only dry storage significantly affected the FG in both species, while flooding and its interaction with dry storage was significant only in *O. rhizomatis* (Supplementary Table S6).

**Figure 3:**
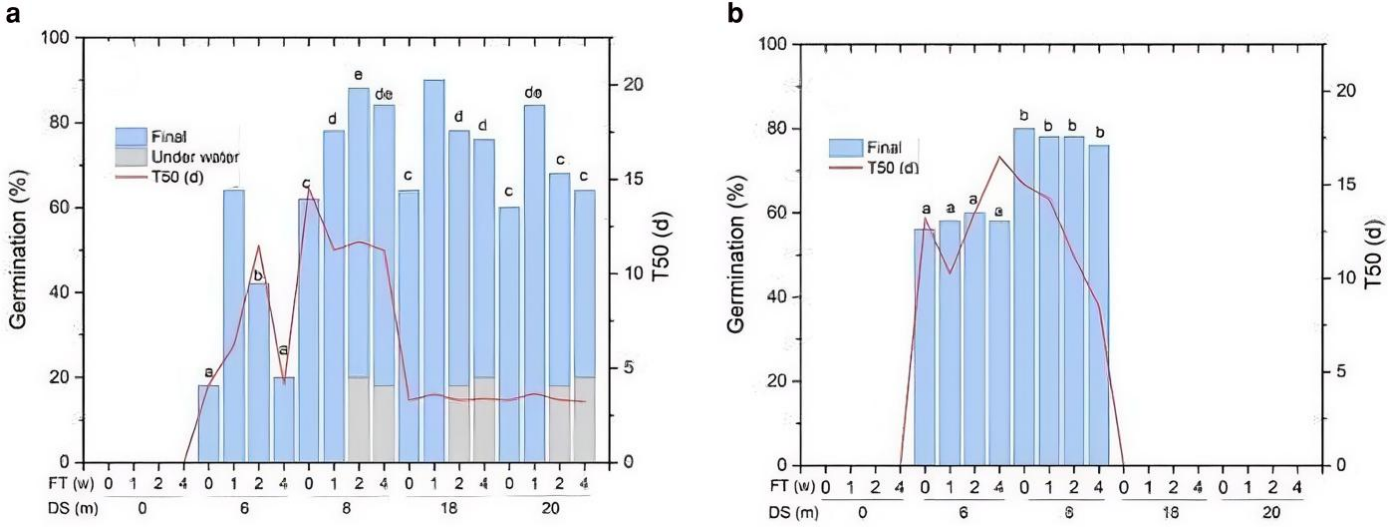
Final germination percentage of *O. rhizomatis* (a) *O. nivara* (b) after 0, 8, 18 and 20 months (m) of dry storage (DS) followed by 0, 1, 2 and 4 weeks (w) of flooding treatments (FT) and incubated on tissue papers moistened with distilled water at 25° C in light/dark (12 hr/12 hr) conditions. Also shown is the fraction of seeds germinating while immersed in water (under water)and the time required to reach 50% germination (red line). Different letters indicate significant differences of germination percentage at *p*< 0.05 based on GLM with Turkey′s post hoc test (Supplementary Table S2 and S3).

When the dry-stored *O. rhizomatis* seeds (for 6, 8, 18, and 20 months) were exposed to a 1-week FT, they had significantly higher germination compared with just dry-stored seeds (Fig. 3a; Supplementary Table S2). Prolonged flooding (i.e. 2–4 weeks) had an overall negative effect on germination percentage, which decreased gradually (except for 8 months dry stored seeds exposed to 2 weeks of flooding). Unlike *O. rhizomatis*, none of the FTs (1, 2, and 4 weeks) improved the germination in *O. nivara*, after any of the dry storage periods when compared with the control (0 week) treatment (Fig. 3b; Supplementary Table S1). Moreover, none of the 18- or 20-month dry stored seeds exposed to flooding (1, 2, or 4 weeks) germinated. The *T*_50_ value of both species decreased significantly with increasing dry storage periods. Furthermore, the *T*_50_ value for the 8-, 18- and 20-month dry storage and 1 week FT significantly differed from the 6-month dry storage and 1 week FT. However, the *T*_50_ of the *O. nivara* seeds exposed to flooding conditions was lower than that of the seeds exposed to comparative dry-storage treatment (Supplementary Table S3).

## Discussion

In this study, we have simulated potential natural dormancy-breaking treatments in a laboratory setting by subjecting seeds of *O. rhizomatis* and *O. nivara* to different durations of dry storage, flooding, or both. Even within a single population of homozygous seeds stored under constant conditions there is a marked variation from seed to seed in period of viability. *O. nivara* and *O. rhizomatis* maintained high control viability values of 0.85 and 0.84, respectively. These closely aligned values provide a reliable baseline for evaluating the effect of dry storage, flooding or both. Seeds of both species were dormant at dispersal as none of them germinated. Seeds of *O. nivara* and *O. rhizomatis* began to germinate after 4 and 6 months of dry storage, respectively. Increasing the periods of dry storage further enhanced the germination percentage in both species (up to c. 85%). These findings highlight that dry storage (dry after-ripening) is required for breaking PD in seeds of both species, consistent with other observations in species that experience dry conditions after seed dispersal (Baskin et al. 2020), including other *Oryza* species (Veasey et al. 2004, Gouda et al. 2025). This adaptation prevents the germination during the dry season (right after seed dispersal), when intermittent rains could elicit seed germination and expose seedlings to a high risk of mortality due to subsequent drought (Escobar et al. 2018). Moreover, the gradual loss of dormancy with the increasing period of dry after-ripening observed here may be a bet-hedging strategy that delays the germination of a fraction of seeds over time to mitigate the risk of reproductive failure. Such strategies appear beneficial in wild plant species adapted to unpredictable conditions, where the high likelihood of both abiotic (e.g. rainfall variability or soil salinity) and biotic challenges (e.g. host-parasite cycles and predation) may result in low seedling survival (Guzzon et al. 2018).

Besides these common germination and dormancy patterns, our results also show some important differences in the response to dry storage between the two species. *Oryza nivara* requires a shorter dry period to break PD compared with *O. rhizomatis.* Variation in the duration of dry storage required to break dormancy has been reported among other wild *Oryza* species. For example, *O. latifolia*, *O. grandiglumis*, *O. eichengeri* and *O. punctata* require only 2 months of dry storage to completely alleviate dormancy (Veasey et al. 2004), while *O. rufipogon* and *Oryza glumaepatula* required 4–6 months of dry storage (Veasey et al. 2004). These dormancy variations among species may be related to the specific habitat conditions of each species, which synchronizes the germination with the prevailing most favourable period for seedling recruitment. In our study species, the overall shorter drying period needed to trigger seed germination in *O. nivara*, is consistent with its wetter microhabitat. Excessively long dry seasons could even constrain plant regeneration from seeds in *O. nivara*. Indeed, unlike seeds of *O. rhizomatis,* which could also germinate after a long period of drying (8–12 months), seed germination of *O. nivara* was null after prolonged drying (>18 months), indicating either a loss of seed viability or an induction of secondary dormancy in this species (Fig. 2).

Our results also show that post-dispersal drying followed by flooding can alleviate PD in *O. rhizomatis*. For example, a combination of 6 months of drying followed by flooding increased germination from 18% (no submergence) to 64% (1-week submergence). Despite this, fresh seeds exposed to flooding did not germinate, indicating that flooding alone cannot alleviate seed dormancy. Instead, a period of drying followed by flooding is necessary to break dormancy in *O. rhizomatis*, with seed germination varying depending on both the duration of dry storage and flooding (Supplementary Table S6). These results suggest that different stages of post-dispersal seeds can sense and respond to varying environmental conditions (drying and flooding) that maximize their chances of successful germination and establishment. Floodwater affects seed dormancy and germination differently among flood-prone species. In *Oryza* species anaerobic germination tolerance is an important trait that has been observed in *O. sativa* (Magneschi et al. 2009) and *Oryza meridionalis* (Wurm 1998). In our species, only a small proportion of seeds germinated under flooding conditions (c. 20%) and only in *O. rhizomatis*. Flooding had no effect on seed germination of *O. nivara*, even after subsequent aerobic exposure, but it promoted dormancy release in *O. rhizomatis*, suggesting that its effectiveness as a dormancy-breaking treatment differs among *Oryza* species. Prolonged flooding (>1–2 weeks) caused an overall reduction of seed germination in *O. rhizomatis*, while it had no effects on *O. nivara*. These results indicate either an induction of secondary dormancy or loss of viability in *O. rhizomatis* seeds caused by prolonged flooding, rather than drying, as shown by *O. nivara.* Unfortunately, despite non-germinated seeds of both species being viable as shown by the cut test, we cannot provide further evidence supporting the presence of dormancy cycling.

The contrasting dormancy pattern observed in our *Oryza* species may be linked to their distinct geographical and ecological distributions. Germination traits are likely to play a significant role in plant distribution, as successful germination is often closely associated with specific habitat conditions (Donohue 2005). In this context, unlike *O. rhizomatis*, which is normally found in areas exposed to periodical short-term flooding, *O. nivara* lives in areas with shallow waters and, therefore, it normally experiences longer floods (Liyanage et al. 2002). This may explain why *O. nivara* exhibits traits that prevent dormancy from being broken by flooding and/or germination under submerged conditions, allowing germination to occur only after aerobic conditions are re-established. On the other hand, brief flooding breaks the dormancy of *O. rhizomatis*, with some seeds even germinating underwater. However, prolonged flooding reinduces seed dormancy in *O. rhizomatis*, potentially signalling unfavourable environmental conditions for subsequent growth of this species. This is a novel and intriguing observation, as previous studies on the effects of flooding in *Oryza* and other species have primarily focused on germination and seed development, while its impact on dormancy has been largely overlooked.

The species-specific dormancy responses observed in this study also have important ecological implications for population persistence and community dynamics in fluctuating wetland ecosystems. Differences in dormancy-breaking requirements between *O. nivara* and *O. rhizomatis* suggest that each species is finely tuned to the hydrological regime of its native habitat, allowing germination to occur only when the probability of seedling establishment is maximized. This study confirmed that seed dormancy and germination are highly adaptive traits (Donohue 2005), showing strict habitat-related behaviour across the two target species. However, climate change could disrupt the harmony between environmental conditions and germination behaviour. Seed dormancy and germination are the life stages that are most sensitive to variation in climate conditions (Walck et al. 2011) and represent a major bottleneck to species recruitment (Rosbakh and Poschlod 2015). Therefore, climatic alterations will undoubtedly affect recruitment of plant regeneration from seeds, as already documented in some cases (Walck et al. 2011). In Sri Lanka, climate change is causing high rainfall variability, inducing extreme floods and droughts (Esham et al. 2018). Due to climate change, the number of extreme rainfall days is increasing at most locations, indicating that the overall intensity of the rainfall is also increasing (Jayawardena et al. 2018). These changes will affect flooding patterns and, in turn, the germination ecology of rice species (Ismail et al. 2013). For example, according to our results, with shortening of the dry season seeds may remain partially dormant, especially in species that require a prolonged dry period after ripening, such as *O. rhizomatis*. Recruitment success of this latter species may further be constrained by prolonged flooding, since it can negatively impact seed germination through an induction of secondary dormancy or loss of viability. Consequently, mitigatory actions such as maintaining appropriate water levels and/or long-term *ex situ* conservation in gene banks will be essential to guarantee the success of conservation efforts.

Beyond their ecological significance, these findings also have practical implications for the conservation and utilization of wild rice genetic resources. Understanding the environmental conditions required to alleviate dormancy can improve protocols for seed storage, regeneration, and germination in *ex situ* conservation programs and gene banks. Our results also highlight that both species have important, yet different adaptations to both flooding and dry conditions, making them a valuable genetic resource for breeding programs. By incorporating genes from *O. rhizomatis*, breeders can develop rice varieties that are more resilient and capable of germinating effectively under or after long periods of hypoxic/dry conditions, as already carried out in other *Oryza* species (Jena 2010).

## Conclusion

*Oryza nivara* and *O. rhizomatis* seeds were dormant at dispersal, and germination percentage was increased with increasing duration of the dry storage period. Drying followed by short-term flooding further alleviated PD in *O. rhizomatis*, whereas it had no effects on *O. nivara.* Prolonged dry storage (>18 months) reset the germination in *O. nivara*, while it had minor effects in *O. rhizomatis.* In contrast, prolonged flooding (>2 weeks) reduced the germination in *O. rhizomatis* but had no effect in *O. nivara*. Differences in the germination responses to drying and flooding between the *Oryza* species investigated here may be related to the diverse environmental conditions of their microhabitats, primary drying and flooding durations, contributing to optimize successful seed germination and seedling establishment.

## Supplementary Material

plag034_Supplementary_Data

## Data Availability

Data available at: https://github.com/Thasajini/DS/blob/main/Dry%20storage-%20germination%20percentage.xlsx. https://github.com/Thasajini/dry-storage-G-/blob/main/Dry%20storage%20%2B%20flooding%20-%20germination%20percentage.xlsx
