# Supplementary material for: Comparative seed dormancy and germination responses to drying and flooding in two Sri Lankan wild rice species
Source: AoB Plants. 2026 Aug 11;18(4):plag034. doi: 10.1093/aobpla/plag034 (PMC13458942; doi:10.1093/aobpla/plag034)
Supplement: Supplementary file 1 [file plag034_supplementary_data.docx]

Supplementary figure 1: Average monthly precipitation and minimum–maximum temperature in Puttalam district, Sri Lanka in 2023. (Climatic data gather from Weather and climate.com)

Precipitation (mm)

Temperature (oC)

| Month | Precipitation |
| --- | --- |
| Jan | 50 |
| Feb | 40 |
| Mar | 55 |
| Apr | 140 |
| May | 138 |
| Jun | 90 |
| Jul | 50 |
| Aug | 55 |
| Sep | 110 |
| Oct | 230 |
| Nov | 260 |
| Dec | 145 |

|  | Minimum temperature | Maximum temperature |
| --- | --- | --- |
| Jan | 23.5 | 30.5 |
| Feb | 24 | 32 |
| Mar | 25.5 | 33 |
| Apr | 26.25 | 32.5 |
| May | 27 | 32 |
| Jun | 26.5 | 31 |
| Jul | 26.25 | 31 |
| Aug | 26 | 31 |
| Sep | 26 | 31 |
| Oct | 25.5 | 30.5 |
| Nov | 25 | 30.5 |
| Dec | 24 | 30 |

Precipitation (mm)

Temperature (oC)

| Month | Precipitation |
| --- | --- |
| Jan | 70 |
| Feb | 30 |
| Mar | 25 |
| Apr | 65 |
| May | 70 |
| Jun | 30 |
| Jul | 40 |
| Aug | 55 |
| Sep | 75 |
| Oct | 225 |
| Nov | 325 |
| Dec | 200 |

|  | Minimum temperature | Maximum temperature |
| --- | --- | --- |
| Jan | 25 | 28.25 |
| Feb | 25.5 | 29.25 |
| Mar | 26.5 | 30.75 |
| Apr | 28 | 32 |
| May | 28.5 | 31.75 |
| Jun | 28.25 | 31.25 |
| Jul | 28 | 30.75 |
| Aug | 27.5 | 30.5 |
| Sep | 27.5 | 30.5 |
| Oct | 26.5 | 30 |
| Nov | 26 | 29 |
| Dec | 25.75 | 28.25 |

Supplementary figure 2: Average monthly precipitation and minimum–maximum temperature in Jaffna district, Sri Lanka in 2023. (Climatic data gather from Weather and climate.com)

Supplementary figure 3: Final germination percentage of six, eight, eighteen and twenty months dry stored seeds of *O. rhizomatis* and six and eight month dry stored seeds of *O. nivara* submerged in distilled water for 0, 1, 2 and 4 weeks (black bars). Also shown the fraction of seeds germinating while inside the water (dashed bars). Different letters indicate significant differences of germination percentage at P < 0.05 based on GLMM post hoc test (Supplementary table 2)

**Supplementary table 1**: Results of Generalized linear model for final germination of different dry storage periods for *O. nivara*

| Species | Dry storage period (Month) | P value | Z value |
| --- | --- | --- | --- |
| *O. nivara* | 2 | 1.000 | 0.000 |
| *O. nivara* | 4 | 0.013 | 2.476 |
| *O. nivara* | 6 | 1.610e-06 | 4.797 |
| *O. nivara* | 8 | 7.670e-09 | 5.775 |
| *O. nivara* | 10 | 2.770e-09 | 5.945 |
| *O. nivara* | 12 | 3.670e-10 | 6.267 |
| *O. nivara* | 18 | 1.000 | 0.000 |
| *O. nivara* | 20 | 1.000 | 0.000 |

**Supplementary table 2**: Results of Generalized linear model for final germination of different dry storage periods for *O. rhizomatis*

| Species | Dry storage period (Month) | P value | Z value |
| --- | --- | --- | --- |
| *O. rhizomatis* | 2 | 1.000 | 0.000 |
| *O. rhizomatis* | 4 | 1.000 | 0.000 |
| *O. rhizomatis* | 6 | 0.001 | 3.098 |
| *O. rhizomatis* | 8 | 4.720e-07 | 5.038 |
| *O. rhizomatis* | 10 | 7.670e-09 | 5.775 |
| *O. rhizomatis* | 12 | 1.920e-09 | 6.005 |
| *O. rhizomatis* | 18 | 4.720e-07 | 5.038 |
| *O. rhizomatis* | 20 | 1.080e-06 | 4.876 |

**Supplementary table 3**: T50 value of the (T50) of *O. rhizomatis* and *O. nivara* seeds exposed to different dry storage (ds) and 1 week flooding treatment (ft). Different lowercase and upper case letters indicate the significant difference between the ds and ds+ 1week flooding treatment(ft), respectively, at p< 0.05.

| Treatment | T_50_ *O. rhizomatis* (hr) | P value | T_50_ *O. nivara* (hr) | P value |
| --- | --- | --- | --- | --- |
| 6m ds | 372.00 ± 9.79 a |  | 349.8 ±5.2 e |  |
| 6m ds + 1week ft | 317.00 ± 6 A |  | 278 ± 1.50 E |  |
| 8m ds | 145.00 ± 1.64 b | 0.019 | 316 ± 5.74 f | 0.021 |
| 8m ds + 1week ft | 192.00 ± 3.26 B | 0.019 | 253 ± 3.87 F | 0.021 |
| 18m ds | 78.30 ±0.97 c | 0.019 | - |  |
| 18m ds + 1week ft | 86.75 ±1.22 C | 0.017 | - |  |
| 20m ds | 78.25 ±1.8 d | 0.018 |  |  |
| 20m ds + 1week ft | 80>00 ±1.15 D | 0.013 |  |  |
|  |  |  |  |  |

**Supplementary Table 4:** Statistics of the generalized linear mixed effect models for the effect of species, dry storage and their interaction on final germination.

| **Factor** | **Chi-square value (χ^2^)** | **Degrees of freedom (df)** | **P value** |
| --- | --- | --- | --- |
| Species | 302.01 | 1 | **0.004** |
| Dry storage | 309.92 | 8 | **< 0.001** |
| Species × Dry storage | 44.70 | 8 | **< 0.001** |

**Supplementary Table 5:** Generalized linear mixed effect models for the effect of species, dry storage, flooding time and their interaction on final germination.

| **Factor** | **Chi-square value (χ2)** | **Degrees of freedom (df)** | **P value** |
| --- | --- | --- | --- |
| Species | 345.90 | 1 | **< 0.001** |
| Dry storage | 1239.33 | 4 | **< 0.001** |
| Flooding time | 38.80 | 3 | **< 0.001** |
| Species x Dry storage | 802.51 | 4 | **< 0.001** |
| Species × Flooding time | 29.33 | 3 | **< 0.001** |
| Dry storage × Flooding time | 17.10 | 12 | 0.147 |
| Species × Dry storage × Flooding time | 16.50 | 12 | 0.170 |

**Supplementary Table 6** Generalized linear mixed effect models for the effect of dry storage and flooding on final germination.

| **Factor** | ***O. nivara*** | ***O. rhizomatis*** |
| --- | --- | --- |
| Dry storage | χ^2^ = 1264.10 (4 df)  **P < 0.001** | χ^2^ = 852.90 (4 df)  **P < 0.001** |
| Flooding time | χ^2^ = 0.18 (3 df)  P = 0.980 | χ^2^ = 86.60 (3 df)  **P < 0.001** |
| Dry storage × Flooding time | χ^2^ = 0.62 (12 df)  P ~ 1.00 | χ^2^ = 33.80 (12 df)  **P < 0.001** |
